# Factors affecting the enrolment of men into a community adherence group in Kavango East Region, Namibia

**DOI:** 10.4102/phcfm.v18i1.5115

**Published:** 2026-03-23

**Authors:** Andreas Nampola, Daniel O. Ashipala, Epafras Anyolo

**Affiliations:** 1 Department of General Nursing Sciences, Faculty of Health Sciences and Veterinary Medicine, University of Namibia, Rundu, Namibia; 2 Department of General Nursing Sciences, Faculty of Health Sciences and Veterinary Medicine, University of Namibia, Windhoek, Namibia

**Keywords:** antiretroviral therapy, community adherence groups, factors, HIV infections, humans, men, Namibia, social support

## Abstract

**Background:**

Community adherence groups (CAGs) are an acceptable and effective strategy to provide social support for people living with human immunodeficiency virus (HIV) and acquired immunodeficiency syndrome (AIDS), as well as for receiving their antiretroviral therapy (ART). However, in Namibia, it is estimated that 30% – 50% of men living with HIV are reluctant to access treatment through these groups, and little research exists on the factors affecting their enrolment.

**Aim:**

This study aimed to explore and describe factors affecting the enrolment of men in CAGs in Rundu District, Kavango East Region, Namibia.

**Setting:**

This study was carried out in the Kavango-East region, one of the 14 regions of Namibia.

**Methods:**

This study followed a qualitative approach that utilised exploratory, descriptive, and contextual strategies. The researcher conducted semi-structured, face-to-face interviews with 15 men between 22 years and 56 years of age in Rundu in September 2024 and October 2024. The participants were purposively selected and the data were analysed thematically.

**Results:**

Participants described numerous barriers hindering men from enrolling in CAGs, including stigma, a lack of information, few perceived benefits, and poor continuity of care. Additionally, participants recommended that men-only groups would improve enrolment.

**Conclusion:**

The study’s findings demonstrate that overcoming barriers to men’s enrolment in CAGs is vital for improving access to HIV treatment. Strategies such as anti-stigma campaigns, increased awareness, incentives, and men-only groups are essential for fostering inclusive and effective HIV care for men.

**Contribution:**

This study highlights the barriers to enrolment in CAGs for men living with HIV, particularly stigma and lack of information, while recommending anti-stigma campaigns, increased awareness, incentives, and men-only groups for improved enrolment.

## Introduction

Human immunodeficiency virus (HIV) has claimed millions of lives for over 50 years.^1^ Sub-Saharan Africa accounts for two-thirds of the global burden of the disease.^2^ Antiretroviral therapies (ART) were introduced to improve the quality of life of people living with HIV by lowering their viral loads, improving their immunity, and reducing their chances of contracting opportunistic infections.^3^ Unfortunately, substantial gaps in HIV testing and ART access have led to a failure to achieve the goal of the Joint United Nations Programme on HIV and AIDS (UNAIDS) of diagnosing 90% of all people living with HIV, providing 90% of those diagnosed with ART, and achieving HIV viral suppression for 90% of those being treated.^4^ Effective strategies that promote healthcare engagement and ART adherence are thus needed to achieve the current ‘95-95-95’ goal by 2030.^5^

However, only 34%^6^ of men in sub-Saharan Africa living with HIV are aware of their status and even fewer access ART.^4^ The World Health Organization (WHO) and the UNAIDS have stressed that successful treatment is linked with early diagnosis, the immediate initiation of treatment, and excellent adherence.^1^ Men contribute disproportionately to the gap in achieving the global target of 95% of people living with HIV knowing their status.^2^ As of 2021, approximately 9.8 million men in sub-Saharan Africa tested HIV positive. Although men accounted for only 38% of people living with HIV, they represented 57% of acquired immunodeficiency syndrome (AIDS)-related deaths.^7^ This discrepancy is linked to lower testing rates, late diagnosis, and poor engagement with treatment. Men delay seeking healthcare because of stigma and cultural barriers, pressure to generate income, facilities and providers that are unfriendly to men, and a lack of routine screening services for men, leading to advanced-stage diagnosis and higher mortality rates.^8,9,10^

The WHO now recommends ART for people living with HIV, regardless of their cluster of differentiation 4 (CD4) cell counts or disease status.^10^ Understanding the barriers to initiation of ART and adherence is thus essential.^11^ Several system-level barriers to ART use, including CD4 testing, delays in enrolling in medical care facilities, insufficient quantities of drugs, and a focus of primary healthcare systems on women of childbearing age and children, have been identified, however studies focused on individual level barriers in resource-limited settings are urgently needed.^12^

These individual barriers include long distances to healthcare services, transportation costs, work responsibilities, and family commitments.^13^ According to Mantell,^14^ additional barriers include long waiting times, stigma, discrimination, a lack of social support, and inadequate information. Morbidity associated with immunosuppression makes HIV or AIDS one of the leading causes of death in Namibia, and HIV infection is the reason for most years of life lost. With a population of ~2.5 million people, the total number of people living with HIV in Namibia stands at 215 000 – a rate of approximately 8.5% HIV prevalence.^15^ In 2016, the WHO endorsed the differentiated services delivery model (DSDM), which was adopted in many countries including Namibia with the aim of improving the health-seeking behaviours of people living with HIV in terms of access to treatment through the formation of community adherence groups (CAGs).^16^ The CAGs were introduced as a programmatic response to some of the obstacles patients encounter when accessing treatment and to alleviate overburdened healthcare facilities.^17^ These groups are formed voluntarily by locals who share the same interests and goals.^18^

In Namibia, CAGs generally consist of 6–12 people who meet certain criteria, such as being an adult, being on ARTs for more than 12 months, and being virally suppressed in two consecutive tests with less than 50 viral load (VL). In the absence of VL, a client must have a rising CD4 cell count or a CD4 count above 350 cells/mm^3^, as well as no other infections, such as Tuberculosis (TB).^19^ The key goals are to give adherence, psychological and financial support, to provide solidarity, and to refill medications.^16,18^ Each group has two supervisors who visit group members monthly at their respective houses to screen them for opportunistic infections such as TB, and they also visit HIV clinics twice a year to pick up the group’s antiretroviral medications (ARVs) on behalf of their group members. Each member is required to visit a health facility once a year for a clinical assessment.^19^

Ensuring that antiretroviral care services are accessible is an important way to improve access to HIV care and adherence to ARVs. Namibia adopted and implemented the DSDM in 2019, which included health facility and community-based delivery models. A recent health survey indicated that out of 1 001 441 men in Namibia aged 15 years and above, only 90 398 (9.02%) consulted health facilities for HIV testing in 2021, compared to 246 872 (14.93%) women. Furthermore, of the 4269 men who tested positive for HIV, only 3470 (81.3%) men opted to start ART.^20^ Surprisingly, only 62 940 (92.2%) men aged 15 years and above who were living with HIV and knew their status were receiving ART, compared to 126 552 (97.9%) women in the same age range.^18^ According to the survey, gaps in HIV diagnosis continue to exist among males aged 15 years and above who are sexually active.^21^ Despite these efforts that include advancing treatment through ART and new long-acting options, improving prevention with methods such as Pre-Exposure Prophylaxis (PrEP) and a focus on vaccine development, expanding testing and diagnosis to increase knowledge of status, implementing strong community-led approaches, fostering global partnerships and strategic alliances to scale up services, and creating supportive environments with HIV-sensitive social protection, however, male enrolment in CAGs as a method to boost adherence to ART remains critically low. Although there is a paucity of research on the low enrolment of men in CAGs in Namibia, the annual report of the Kavango East Regional Health Directorate found that CAGs enrolment in Rundu district in 2023 was 234 men and 812 women.^22^ In 2024, 222 men were enrolled compared to 1003 women.^23^ Given that the catchment population is 63 430 and the number of patients on ARV is 11 747, this indicates that there is a substantial risk that people are not adhering to treatment. These low levels of male enrolment in CAGs in Rundu imply that there are barriers to enrolment in CAGs, thus this study aimed to explore and describe the barriers affecting the enrolment of men into CAGs in Rundu. By identifying these barriers, the research sought to inform targeted interventions and ongoing strategies that can be used to increase and maintain sustained enrolment among men in CAGs.

## Research methods and design

This study followed a qualitative approach utilising exploratory, descriptive and contextual strategies. Exploratory research was applied, and provided insight into the factors affecting the enrolment of men into CAGs. In addition, the barriers that prevent men to join the CAGs remain insufficiently understood. It is therefore important to explore these factors to gain deeper insights into the nature and extent of their barriers. Moreover, the study was of a descriptive design as it provides accurate descriptions and in-depth insight into men’ real-life viewpoints, which can be useful to address a problem under study.^24^ Descriptive research is employed to explore and describe phenomena within real-life contexts, to gain an in-depth understanding that can inform the barriers that prevent men to become CAGs members. The study was contextual as the interviews with CAGs men were conducted with CAG groups in Rundu district, a setting where CAG members are and an environment that directly shapes their experiences of factors affecting men’s low enrolment into CAGs. Conducting the research in this natural setting helped to ensure the collection of valid and accurate data, minimally influenced by external factors. This approach is particularly relevant in the Namibian context, where cultural beliefs, social structures, economic constraints, and infrastructural limitations uniquely affect men’s experiences and differ significantly from those in other parts of the world.

This design enabled researchers to explore how the respondents understood their experiences, understandings and cultural and environmental or structural factors surroundings related to the use of CAGs.^25^

### Setting, population and sample

The research was conducted in Rundu, a key urban Centre in the Kavango East Region of Namibia, with an approximate population of 63 000 residents. The antiretroviral services offered by public health services in Rundu include pre-counselling to HIV testing, post-counselling prior to the disclosure of results, initiation to antiretroviral treatment upon testing positive for HIV, health education, and initiation into a CAG after a year of treatment. The ART clinics include registered nurse managers who coordinate other staff, registered nurses, enrolled nurses who prescribe medications, Total Control of Epidemic (TCE) members, health extension workers or community nurses who largely do fieldwork, and doctors who are predominantly based at the Rundu State Hospital. Nurses offer more facility-based ART services than the TCE and health extension nurses, who offer more community-based ART services. The CAG is comprised of a minimum of 6–12 maximum ART stable patients with no complicated regime, 1 year or more on HIV treatment, 40 copies of viral loads, no history of alcohol or drug abuse, not breastfeeding and not pregnant as criteria for enrolment.

The inclusion criteria for this study were: being a resident of Rundu, male living with HIV, aged between 18 years and 60 years, and being willing to participate by signing written consent. A purposive sampling method was employed, resulting in the selection of 15 participants from various CAGs to ensure individual in-depth experiences and perspectives.

Prior to data collection, the researcher obtained approval to conduct the study from the nurse managers as gatekeepers of clinics in Rundu, primary healthcare officials, Centers for Disease Control and Prevention (CDC) located at Rundu Intermediate Hospital, the Kavango East Regional Council, the constituency councillor for Rundu, and the village headman. Participants were recruited using purposive sampling.^26^ The primary method of recruitment involved study information flyers, which were posted by the researcher on the notice boards of selected clinics and hospitals with permission from supervisors, managers, and unit managers. These flyers included the researcher’s contact details and invited interested individuals to attend an information session, where the purpose of the study was explained and questions about participation were addressed. In addition to this passive recruitment, potential participants in distant rural villages were contacted by phone by clinic staff who requested their permission from the men who they contacted to share their contact information with the researcher to enable them to be contacted, and the researcher directly explained the study and addressed their questions. Individuals who expressed an interest in participating and met the inclusion criteria were scheduled for interviews, which were arranged to coincide with their next clinic follow-up visit. At that time, verbal and written consent were obtained before conducting the interviews. Recruitment continued until data saturation was reached, with a total of 15 participants interviewed.

### Data collection

Data for this study were collected between September 2024 and October 2024, with a second researcher conducting all interviews. Participants were required to provide written consent before taking part in the study and were assured of their right to withdraw at any time. An interview guide was developed based on the research questions, literature review, and study objectives. The semi-structured interviews followed this guide to explore why men in Rundu were not enrolling in CAG. Participants were offered the option to be interviewed in English, Oshiwambo, or Rukwangali, languages commonly spoken by the majority ethnic groups in Rundu, however all participants preferred to be interviewed in English at venue convenient to participants. Each session lasted between 50 min and 60 min. With prior consent, the researcher used an audio recorder while also taking notes.

A pilot study was conducted with three participants from various villages in Rundu to test the relevance and usability of the interview guide. The pilot participants met the same criteria as those for the main study. The interview guide was modified slightly based on insights gained during the pilot interviews, including the addition of new questions and the combination of overlapping ones. Data from the pilot interviews were not included in the final analysis. The following questions were posed during the interviews:

- *What is your understanding of CAGs?*
- *What factors affect the enrolment of men in CAGs?*
- *In your own opinion, what can be done to increase the enrolment of men into CAGs?*

### Data analysis

The researcher used an inductive approach to analyse the data, using the thematic analysis technique.^27^ The data gathered from the recordings were transcribed verbatim, following which they were analysed using Braun’s six-step method of data analysis.^28^ This included: becoming familiar with the data; generating initial codes; coding data using nine steps; determining and reporting themes; defining and naming themes; and interpreting the data.

### Trustworthiness

The trustworthiness of the study was ensured by using Lincoln and Guba’s model, which confirms the credibility, dependability, confirmability, and transferability of a study.^29^ Credibility was realised by holding pilot interviews; establishing data saturation; prolonging participant engagement; and active participation. Transferability was achieved by incorporating rich descriptions of the perspectives of the participants.^30^ Dependability was ascertained via a peer debriefing, as well as extended engagement and member checking. An inquiry audit using an external reviewer ensured confirmability. Finally, reflexivity was established by the researcher remaining aware of his roles in the study, which included ongoing reflection on personal behaviours and how they might affect the research.^31^ In this study, the research was a full time undergraduate nursing student at a institution of higher education in Namibia at the time of the study. He never worked in the public health sector and he has no ties to it. There was, therefore, no conflict of interest.

### Ethical considerations

Ethical clearance to conduct this study was obtained from the University of Namibia’s Faculty of Health Sciences and Veterinary Medicine/School of Nursing and Public Health Research and Ethics Committee (No. SoNREC 85/2023) and the Ministry of Health and Social Services’ (MoHSS) Institutional Review Board (IRB) (No. 22/4/2/3). Written informed consent was obtained from all participants before participating in the study, including the permission to audio-record their interviews. The participants were assured of their right to opt not to join the study by declining to sign consent or withdraw at any time without penalty. Furthermore, participants were able to decide on the date, place and time of their interview. The possibility existed that some participants might experience discomfort or become emotional during the interviews. Participants were notified of the option to consult a social worker at the Department of Social Welfare should this have been necessary. This sensitivity to the participants’ feelings was aligned with the right to self-determination. The interviewees were assigned codes to ensure their anonymity and confidentiality, and all data were kept on a password-protected computer. These data will be disposed of according to the university’s policy where research data are retained for a minimum of 5 years, and after the 5-year retention period, the data can be discarded according to ‘acceptable environmental standard’.

## Results

### Research participants’ characteristics

Participants were between the ages of 22 years and 57 years, 10 of whom had been involved in CAGs for 3 years to 4 years. All 15 participants were black African people. Nine were employed and 11 were married. Four had only completed primary school and 11 had finished secondary school. Table 1 shows the participants’ demographic data.

**TABLE 1:** Characteristics of study participants.

Variable	Frequency (*n*)
**Age (years)**	
22–30	3
31–39	4
40–48	6
49–57	1
**Years of being in a CAG**	
1–2	5
3–4	10
**Marital status**	
Married	11
Unmarried	4
**Employment status**	
Employed	9
Unemployed	6
**Academic level**	
Primary	4
Secondary	11

CAG, community adherence group.

### Key themes

The three themes that emerged from the data analysis included: personal understanding of the concept of CAGs; perceptions of CAGs as community antiretroviral groups; and recommendations to improve the enrolment of men into CAGs. These themes and sub-themes are presented in Table 2.

**TABLE 2:** Themes and sub-themes.

Themes	Sub-themes
1. Participants’ conceptual understanding of CAGS	1.1 Community antiretroviral refill group 1.2 Community adherence group
2. Barriers hindering men from enrolling in community adherence groups	2.1 Stigma 2.2 Information on CAGs 2.3 Few perceived benefits 2.4 Poor continuity of care
3. Recommendations to improve the uptake of men in CAGs	3.1 Formation of an anti-stigma campaign in the community 3.2 Increase awareness of CAGs 3.3 Use of incentives that will promote more men to join a CAG 3.4 Income generating project 3.5 Formation of men-only CAGs

CAG, community adherence group.

#### Theme 1: Participants’ conceptual understanding of community adherence groups

This theme is a description of the participants’ understanding of the concept of a CAG. The sub-themes that emerged from this theme were community antiretroviral refill groups and CAGs.

**Sub-theme 1.1: Community antiretroviral refill groups:** The study participants mentioned that CAGs are HIV antiretroviral refill groups formed by patients living with HIV who have been on treatment for a long period, who reside in the same community, and have group leaders who collect medication from a health facility and distribute it to others.

One of the participants described it as follows:

‘They are community antiretroviral refill groups which were formed by us with the assistance of TCE and nurses for HIV patients to be treated in groups whereby we go for follow ups together after six months. Groups have a leader that checks up on members and helps to fetch members’ medication.’ (P8, 56 years old, unemployed)

Another participant said:

‘CAG is a HIV medication refill group in a community comprised of both male and female HIV positive patients staying at close community being treated together and with a group leader who collect medication at the clinic and take them to the owners within the same group.’ (P6, 33 years old, unemployed)

**Sub-theme 1.2: Community adherence group:** Under this sub-theme, the participants described that CAGs are those that enable people living with HIV to adhere to their ART treatment:

‘CAGs are community adherence groups whereby HIV patients do more self-management of their care. Groups have a group leader that collect members’ medications and screens them for infections such as TB.’ (P12, 37 years old, employed)

#### Theme 2: Barriers hindering men from enrolling in community adherence groups

This theme contains participants’ descriptions of aspects that prevent or inhibit men from utilising CAGs. The sub-themes are as follows: stigma and confidentiality breaches; a lack of information; a lack of perceived benefits; and a poor continuity of care.

**Sub-theme 2.1: Stigma:** The participants in this study found that some men do not disclose their HIV positive status to their families or friends to prevent mistreatment, to avoid being talked about, to avoid blame, to maintain their social status, and to keep peace in the community. Disclosing one’s status to the group members is necessary to enrol in a CAG:

‘Some men still believe that if they are the only one on antiretroviral treatment at home, they prefer to keep it a secret because if the wife and children hear it, they will mock them, they will not want to share anything with them at home and they also believe that the CAG members will spread the news to the entire community so that they will be labelled as sick people awaiting for death in the community thus they prefer acquiring their treatment privately.’ (P3, 37 years old, employed)

**Sub-theme 2.2: Information on CAGs’:** The study participants reported that most men are not aware of the existence of CAGs, the services offered, the advantages of being enrolled, and the entry criteria prior to enrolment:

‘Not all the men were told about this groups, the services being offered and the advantage of joining the groups such as saving money because we no longer go for follow up every month and we help each other financially and psychologically.’ (P5, 39 years old, unemployed)

**Sub-theme 2.3: Few perceived benefits:** Participants in this study revealed that they perceive there to be minimal benefit to acquiring ART services through CAGs compared to health facilities, which prevents them from enrolling:

‘Some men say community adherence groups are just for gossiping and there is no benefit of joining them, it will just make you to be hated by other community members and beside we are still drinking the same medication, whether in CAGs or not.’ (P1, 46 years old, unemployed)

‘My neighbor says community adherence groups should be for those that stays far from town, for him he stays in town and can have access to ART treatment anytime he decides without any struggle of transport neither money.’ (P4, 44 years old, unemployed)

**Sub-theme 2.4: Poor continuity of care:** Participants suggested that joining CAGs is not an easy decision to make because it is perceived to be associated with poor continuity of care as they are deprived from interacting with professional healthcare providers such as nurses for 6 months at a time. This decreased contact with professional healthcare provider is felt to increase their risk of acquiring HIV-related co-infections such as tuberculosis, as although they are screened every month by their group leaders, they are not really knowledgeable about such issues:

‘My friend said he cannot be treated by a fellow patient just because [*he*] is a group leader, he must come and screen him for TB, it will jeopardise his health and put his status on more risk, he will continue with his health facility-based care.’ (P1, 36 years old, employed)

‘Sometimes the group leader goes to the hospital and comeback without all our medication because they are all not in stock, this put our health at risk because they don’t pass at other health facility to ask if there is those medication in the pharmacy but if I go there myself I can go and ask at other facility where I am not enrolled. This discourage most people from joining.’ (P5, 39 years old)

#### Theme 3: Recommendations and mechanisms for improvement

This theme describes the participants’ suggestions for what needs to be done in order to improve the enrolment of men into CAGs. The sub-themes that emerged from this theme are: formation of anti-stigma campaign; creating awareness about CAGs; use of incentives; creating job opportunities for group members; and formation of men-only CAGs.

**Sub-theme 3.1: Formation of human immunodeficiency virus anti-stigma campaign:** In this sub-theme, the study participants expressed the need for healthcare and government workers to host anti-stigma campaigns around the community:

‘Nurses and total Control of the Epidemic (TCE) people should march around different communities to encourage people living with HIV to open up by disclosing their HIV positive status to their family members and friends.’ (P4, 44 years old, unemployed)

**Sub-theme 3.2: Creating awareness about community adherence groups:** Participants in this study suggested that better marketing about the existence and operation of CAGs should be adopted to ensure that more men living with HIV are aware of them, including all the necessary criteria prior to enrolment:

‘The government and non-governmental organizations that deal with HIV-related issues need to formulate new marketing strategies that will accelerate the enrolment of men into CAGs rather than depending only on nursing outreach which is not that effective. Perhaps we should advertise it through television or radio programmes as well as newspapers.’ (P2, 44 years old, unemployed)

**Sub-theme 3.3: Use of incentives:** Participants expressed that the Ministry of Health and Social Services should implement a strategy of compensating men who are enrolled in a CAG as a mechanism to retain them and attract others:

‘We should be recognized for being in these groups, the Ministry of Health should give us money, food and T-shirts as other ministries do to marginalized people. When men hear that we are now getting all those, they will join the groups.’ (P6, 47 years old, employed)

**Sub-theme 3.4: Income generating project:** The study participants reported that job opportunities should be created for men and women who receive ART through CAGs, because they are required to eat a balanced diet which the majority cannot afford. The participants explained that creating a finance generating project will help them meet and work together to feel a sense of belonging in the group. This will also serve as a facilitator and source of encouragement, which will attract more men to enrol in a CAG:

‘My suggestion is that the government should create job opportunities such as gardening since most of us are unemployed, whereby we will be working in those gardening farms and they will be paying us as well as giving us food from this garden. Obviously if that happens then all the HIV positive patients on treatments will join this groups.’ (P4, 44 years old, unemployed)

**Sub-theme 3.5: Formation of men-only community adherence groups:** In this sub-theme, the study participants expressed the need to create CAGs for men only; only female healthcare workers would be allowed:

‘Nurses should make CAGs for men only. Most of my friends have transferred to health facility where there are men only. Maybe this will encourage my gender to utilize these group services.’ (P10, 40 years old, employed)

## Discussion

The study’s objective was to explore and describe factors affecting the enrolment of men in CAGs in Rundu District, Kavango East Region, Namibia. It highlights the key barriers to enrolment in CAGs for men living with HIV, particularly stigma and a lack of information, while recommending anti-stigma campaigns, increased awareness, incentives, and men-only groups for improved enrolment.

One of the key themes was participants’ understanding of CAGs, with the interviewees describing them as antiretroviral refill groups formed by patients living with HIV, who have been on treatment for a long period, who reside in the same community, and who have group leaders that collect medication from a health facility and distribute it to them. The participants believed that CAGs play a significant role in improving access and adherence to HIV treatment by reducing the need for frequent healthcare facility visits. The model fosters peer support, enhances adherence, and minimises treatment interruptions through group leadership and 6-month follow-ups. Similar models across Africa have led to improved adherence and viral suppression.^32,33^ Uganda’s experience highlighted the economic benefits of CAGs by reducing travel costs and time lost from work,^34^ however challenges such as stigma, confidentiality concerns, and group cohesion issues persist, emphasising the need for ongoing support from healthcare providers and policymakers to strengthen and sustain these models.^35^ Participants also found that CAGs enable people living with HIV to access their ART in rural communities by reducing clinic visits. They also stated that CAGs promote self-management, with leaders collecting medication and screening for infections like TB. This model enhances adherence, ensures early disease detection, and fosters peer support, easing healthcare burdens and improving patient outcomes. These findings echo those of a study conducted in Uganda by Kasande et al.^36^, who described CAGs as groups formed by patients living with HIV who reside in the same rural communities, which are created to reduce congestion at health facilities, promote meaningful involvement, and enhance self-care management.

In addition, the study also highlighted aspects that prevent or inhibit men from utilising CAGs such as stigma and confidentiality breaches; a lack of information; a lack of perceived benefits; work commitments; and poor continuity of care. Stigma and fear of discrimination keep men from openly seeking HIV treatment, leading them to avoid community groups and travelling far for medication to avoid isolation and judgement. This fear of being blamed or labelled reinforces secrecy, which hinders treatment adherence support. This aligns with earlier studies on the facilitators of, and barriers to, men engaging in CAGs.^37^ Stigma is a barrier as there is a perception that people living with HIV are promiscuous, which correlates with a study conducted in Zimbabwe by Mantell.^14^ A study conducted by Hlongwa et al.^37^ in South Africa on barriers and facilitating factors to HIV treatment among men also revealed that most avoid joining HIV-related programmes for fear of being stigmatised. Furthermore, a Ugandan study by Muzeyi et al.^13^ discovered that men were not comfortable disclosing their HIV status to others, and thus opted to access their ART elsewhere.

Some participants mentioned that lack of awareness of, and information about, CAGs was also highlighted, as one of the factors that led to the low enrolment of men in CAGs. Numerous participants were found to be unaware of benefits such as reduced follow-ups and financial and psychological support. These findings are consistent with research conducted in Zimbabwe.^38^ According to Mantell,^14^ a lack of awareness is a major contributing factor, as most men stress that they are not aware of CAGs. An exploratory study conducted in sub-Saharan Africa by Coursey et al.^39^ on the unique barriers and facilitators that affect men when it comes to HIV care found that men lack more awareness than women because of work commitments and not wanting to be associated with healthcare facilities. In addition, an exploratory study^12^ that investigated barriers to the implementation and scaling of differentiated service delivery models for HIV treatment in Africa showed that excellent outreach is necessary to ensure that men are reached.

Participants in this study argued that there are fewer benefits to acquiring ART services through CAGs compared to health facilities, which prevents them from enrolling. Some participants thus see CAGs as offering little benefit beyond priority treatment, with some men viewing them merely as gossip platforms that may cause social stigma. Others believe CAGs are only useful for those in remote areas; urban residents find them unnecessary because of easy ART access. Enhancing CAGs’ benefits could therefore improve participation, which is as per the findings of Nyato et al.^40^ Kasande et al.^36^ also report that poor participation of men in client-led ARV refill groups is because of the perception that there are few benefits of being a CAG member, apart from longer-term refills and reduced transportation costs. Some participants in this study complained that joining CAGs is not an easy decision to make because they perceive it to be associated with poor continuity of care as they are deprived from interacting with professional healthcare providers for 6 months at a time. This, they believe, puts them at risk of acquiring HIV-related co-infections such as tuberculosis, as although they are screened every month by their group leaders, they argue that they are not really knowledgeable about such issues.

Medication shortages were also highlighted in this study as one of the factors discouraging the participation of men, especially if group leaders fail to seek alternatives at other facilities, which individuals might do. These findings echo those of Office et al.^41^, as well as Kushemererwa et al.^38^ in western Uganda, in which participants disclosed that transferring from facility-based care to community-based care would put them at risk of contracting opportunistic infections. Furthermore, research conducted in rural eSwatini found that the majority of patients prefer accessing their treatment at a health facility as it provides them with more information related to their illness as they are interacting with professional ART service providers.^40^

The last key theme of the study provided suggestions for what needs to be performed in order to improve the enrolment of men into CAGs. Participants expressed the need for healthcare and government workers to host anti-stigma campaigns, including public awareness efforts to encourage disclosure, promote acceptance, and prevent discrimination, as strengthening community support can help ease the burden faced by people living with HIV. Past research by Mantell et al.^14^ discovered that minimising stigma through different strategies such as campaigns encourages more men living with HIV to disclose their status to their family members, friends, and society.^14^ Omona and Kanyerezi^42^ suggested that ARV clinic healthcare workers should support people living with HIV to consider disclosing their HIV status to relatives as soon as possible, as a lack of disclosure hinders men from enrolling into CAGs. Furthermore, Ndirangu et al.^43^ suggested that more health talks should be given to prepare patients psychologically to help them to disclose their HIV status. They added that outreach campaigns should be hosted to alleviate the stigma and discrimination among community and family members.

Some participants in this study believed that marketing would lead to the successful enrolment of men into CAGs. They argued that healthcare workers should adopt effective marketing strategies such as outreach services, newspaper articles, and TV and radio advertisements. This recommendation is in line with those of other researchers such as Coursey et al.^39^, who suggested that healthcare workers should render quality health education at ART clinics and ask men who are already part of a CAG to invite others to join. The findings of this study on a proposed strategy for the best implementation of CAGs and closing the information gap is in line with those of Kushemererwa et al.^38^, who found that providing information so men are aware of all available services and advantages will have a positive outcome on enrolment.

The participants also suggested the implementation a strategy of compensating men who are enrolled in a CAG by the Ministry of Health and Social Services as a mechanism to retain them and attract others. Other researchers found the same views, for example, in Uganda, Omona and Kanyerezi^43^ agreed that members should be given gifts such as T-shirts, trousers, and food as a mechanism to motivate and retain them. Ndlovu et al.^44^ similarly recommended that CAGs members should receive donations in the form of money, food, T-shirts, and bicycles. Job opportunities should also be created for men and women who receive ART through CAGs, because they are required to eat a balanced diet which the majority cannot afford. The participants explained that creating a finance generating project will help them meet and work together to feel a sense of belonging in the group. This will also serve as a facilitator and source of encouragement that will attract more men to enrol in a CAG. The suggestion of creating income generating projects for people living with HIV has been corroborated by other researchers. Hlongwa et al.^37^ suggested that if beneficial opportunities such as employment are made available to all consistent HIV programme participants, it will serve to attract others and mitigate some of the barriers to enrolment. In addition, studies have found that economic activity would enhance members’ sense of belonging and would also work as a cover for those who did not wish to disclose their HIV status.^43^

Finally, participants expressed the need to create CAGs for men only as some men prefer gender-specific CAGs, citing conflicts and lack of confidentiality in mixed-gender groups. A number of participants believed that forming male-only groups would encourage participation and improve service utilisation. Some men had transferred to facilities with male-only groups, emphasising the need for healthcare workers to accommodate this preference. Early research conducted in Malawi by Phiri et al.^45^ similarly highlighted that health facilities should form men-only groups because men are perceived to be more likely to open up and disclose their HIV positive status to other men. A similar study conducted in Zambia found that men’s groups are more likely to be free from stigma, confidentiality breaches and arguments, which creates harmony.^46^

### Strengths and limitations

A comprehensive and nuanced knowledge of the obstacles to men’s enrolment in CAGs in Rundu was made possible by interviewing 15 participants to achieve data saturation. However, even though the qualitative results provide in-depth contextual information, it is possible that they cannot be applied to a wider range of participants. It could have been more compelling if some men who had not joined CAGs had participated. Purposive sampling made it possible to choose people with pertinent experience, but this method may have created bias because the participants might not accurately reflect the larger population of males living with HIV in the area. Furthermore, social desirability bias may have resulted from the use of self-reported information obtained through interviews, namely, the participants may have given answers in a way they felt was more acceptable to the researcher. Triangulating data with viewpoints from medical professionals and other HIV care stakeholders would help the study’s conclusions be more legitimate and holistic.

## Conclusion

The findings of this study indicate that the participation of men in CAGs is hindered by various obstacles, such as stigma, a lack of awareness, a lack of perceived benefits, and worries around confidentiality. The study’s findings highlight that men who joined CAGs benefit through emotional support, personal growth, and practical assistance. These benefits help to counter feelings of isolation and improve overall well-being by creating a safe environment where men can connect with peers facing similar issues. The results highlight the potential value of capitalising on social support, particularly among men by encouraging disclosure. Thinking in terms of support at multiple levels, there is value in stimulating men’s ability to address anticipated stigma, but this may need to be tailored for the different life circumstances of men, undergirding existing emotional and instrumental support, and providing additional structures to increase missing informational support. The study’s findings call for well-articulated plans and actions from healthcare professionals, community leaders, and support networks to tackle the issues that men encounter in accessing CAGs. The findings from this research are vital for policymakers and the Ministry of Health and Social Services (MoHSS), as they highlight the significance of focused interventions and educational initiatives aimed at addressing stigma related to and fostering understanding and acceptance of CAGs. By tackling these obstacles, the MoHSS can devise strategies that not only boost enrolment, but also maintain ongoing participation in CAGs, thereby enhancing health outcomes for men living with HIV in the area. The findings from this study can provide a basis for creating impactful programmes that encourage men to become involved with CAGs and improve their adherence to treatment.
